# Sarcomatous intrahepatic cholangiocarcinoma: Case report and literature review: Erratum

**DOI:** 10.1097/MD.0000000000012951

**Published:** 2018-10-19

In the article, “Sarcomatous intrahepatic cholangiocarcinoma: Case report and literature review”^[1]^ which appeared in Volume 97, Issue 39 of *Medicine*, the last line of Table 1 appeared incorrectly. The corrected table should be:

**Table 1:**
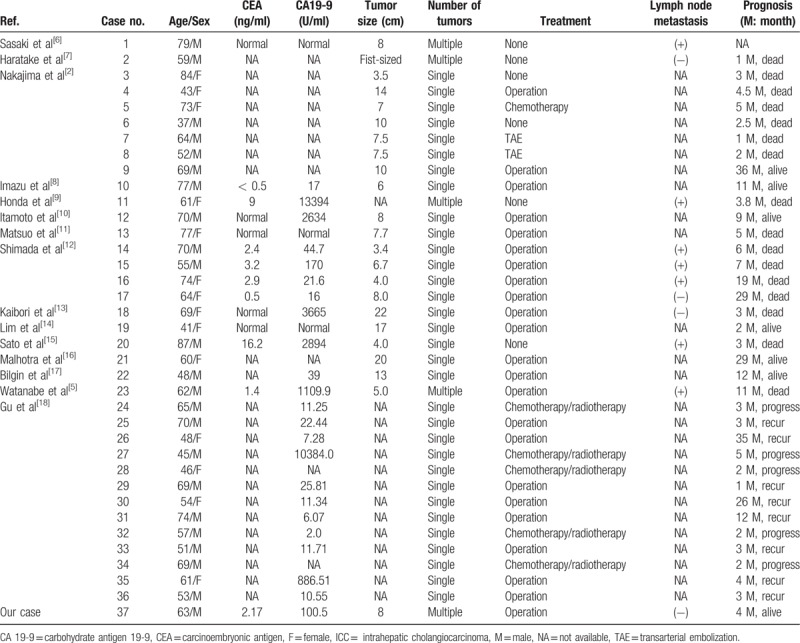
Summary of sarcomatous ICC reported in the English-language literature.
